# A Human‐Computer Interaction Strategy for An FPGA Platform Boosted Integrated “Perception‐Memory” System Based on Electronic Tattoos and Memristors

**DOI:** 10.1002/advs.202402582

**Published:** 2024-07-24

**Authors:** Yang Li, Zhicheng Qiu, Hao Kan, Yang Yang, Jianwen Liu, Zhaorui Liu, Wenjing Yue, Guiqiang Du, Cong Wang, Nam‐Young Kim

**Affiliations:** ^1^ Shandong Provincial Key Laboratory of Network Based Intelligent Computing School of Information Science and Engineering University of Jinan Jinan 250022 China; ^2^ School of Integrated Circuits Shandong University Jinan 250101 China; ^3^ School of Space Science and Physics Shandong University Weihai 264209 China; ^4^ School of Electronics and Information Engineering Harbin Institute of Technology Harbin 150001 China; ^5^ RFIC Centre Department of Electronics Engineering NDAC Centre Kwangwoon University Seoul 01897 South Korea

**Keywords:** electronic tattoo, FPGA platform, human‐computer interaction, integrated system, memristor

## Abstract

The integrated “perception‐memory” system is receiving increasing attention due to its crucial applications in humanoid robots, as well as in the simulation of the human retina and brain. Here, a Field Programmable Gate Array (FPGA) platform‐boosted system that enables the sensing, recognition, and memory for human‐computer interaction is reported by the combination of ultra‐thin Ag/Al/Paster‐based electronic tattoos (AAP) and Tantalum Oxide/Indium Gallium Zinc Oxide (Ta_2_O_5_/IGZO)‐based memristors. Notably, the AAP demonstrates exceptional capabilities in accommodating the strain caused by skin deformation, thanks to its unique structural design, which ensures a secure fit to the skin and enables the prolonged monitoring of physiological signals. By utilizing Ta_2_O_5_/IGZO as the functional layer, a high switching ratio is conferred to the memristor, and an integrated system for sensing, distinguishing, storing, and controlling the machine hand of multiple human physiological signals is constructed together with the AAP. Further, the proposed system implements emergency calls and smart homes using facial electromyogram signals and utilizing logical entailment to realize the control of the music interface. This innovative “perception‐memory” integrated system not only serves the disabled, enhancing human‐computer interaction but also provides an alternative avenue to enhance the quality of life and autonomy of individuals with disabilities.

## Graphical abstract

This study proposes an FPGA‐based platform‐boosted integrated “perception‐memory” system that combines electronic tattoos and memristors to sense, recognize, and memorize physiological signals. The most valuable contribution of this work is that the integrated system enables the manipulator to follow the human hand, emergency call, and smart home functions, which are capable of assisting people with disabilities to live autonomously and conveniently.

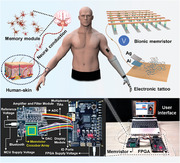

## 1 Introduction

With the increasing demand for the cause of individuals with disabilities,^[^ ^1^, ^2^, ^3^, ^4^ ^]^ enhancing the quality of life and promoting autonomy have become one of the key researches on wearable devices. To date, most wearable devices in the market are based on rigid silicon substrates, which makes it difficult to form a conformal interface with the skin. Due to the tremendous merits of its simple structure and comfortable wearing,^[^ ^5^, ^6^, ^7^, ^8^ ^]^ the flexible sensor that tightly adheres closely to human skin and accurately perceives biological physiological signals has become a desirable component of wearable electronic devices.^[^ ^9^, ^10^, ^11^, ^12^ ^]^ Currently, the majority of physiological signal detection relies on commercial flexible wet gels (Ag/AgCl electrodes), which have the property of easier drying after prolonged use, resulting in poor signal quality and motion artifacts. In contrast, the ultra‐thin, flexible, and stable characteristics of electronic tattoos have distinct assets in monitoring human physiological signals and can be widely applied in fields such as human‐computer interaction and intelligent prosthetics.^[^ ^13^, ^14^, ^15^, ^16^ ^]^ Hence, an increasing number of researchers are focusing their attention on electronic tattoos. For instance, Zhang et al. proposed a healable and multifunctional electronic tattoo based on a graphene/silk fibroin/Ca^2+^ combination, which responds to environmental changes such as strain, humidity, and temperature, making the electronic tattoo highly sensitive to a variety of stimuli.^[^ ^17^ ^]^ Further, a novel type of electronic tattoo based on chemical vapor deposition large area of Mo_2_C film was realized by Liu and workers, which was applied to accurately and imperceptibly capture body electrophysiological signals and interface with robots.^[^ ^18^ ^]^ At present, most of the reported studies mainly focus on the promotion of ultra‐thin and skin sticking,^[^ ^19^, ^20^, ^21^ ^]^ but meeting the needs of low‐cost, facile preparation, and stability is still filled with challenges. Therefore, developing a new comfortable electronic tattoo to solve the above‐mentioned issues is essential.

**Figure 1:**
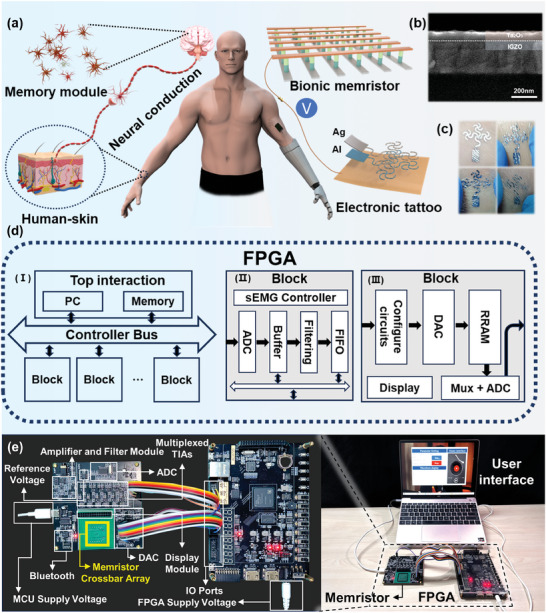
Bionic system and the presentation of FPGA platform. a) Schematic illustration of a biological system with physiological signals generation and memory. b) SEM image of the Ta_2_O_5_/IGZO/ITO structure. c) Presentation of the electronic tattoo. d) Block diagram of FPGA‐based control system. e) Photograph of the hardware system, consisting of an FPGA controller loaded with an ADC, two DACs, multiplexed TIAs, a memristor array, a Bluetooth module, and an sEMG signal amplification and filtering module.

Previous research has mostly just tried to sense the biological signals of the body, which significantly restricts the application scenarios in everyday life.^[^ ^22^, ^23^, ^24^ ^]^ Combining the obtained actions during the sensing process with the brain, and memorizing the action information, as shown in **Figure** **1**a (left), contributes to overcoming the limitations of a single sensor and immensely enhances information interaction. Unfortunately, a large proportion of reports only show solicitude for one aspect of perception, recognition, or memory,^[^ ^25^, ^26^, ^27^, ^28^ ^]^ and the bionic sensor control system that is capable of simultaneously achieving the above functions has few reported. What's worse, given the high cost and the limitation of the manufacturing process, the development of traditional memory is approaching a bottleneck, which restricts the enhancement of chip performance.^[^ ^29^, ^30^, ^31^ ^]^ To advance memory technology, researchers have proposed various types of late‐model electronic devices. Owing to its non‐volatile and unique charge transfer characteristics, the memristor has grown into one of the current research hotspots,^[^ ^32^, ^33^, ^34^ ^]^ which has the superiority of in‐memory computing and low power consumption.^[^ ^35^, ^36^, ^37^, ^38^, ^39^, ^40^ ^]^ For example, Yan et al. proposed the digital design of an Ag/HfO_2_/black phosphorus/Pt bilayer structure on a silicon substrate, and the device exhibited stable state switching and centralized resistance distribution characteristics, along with good retention and durability during testing.^[^ ^41^ ^]^ More importantly, memristors have been widely used in simulating biological memory and computing due to their natural two‐terminal structure, which closely resembles biological synapses.^[^ ^42^, ^43^, ^44^ ^]^ For instance, Liu et al. reported a CeO_2_/Nb‐SrTiO_3_ heterojunction memristor that closely mimics biological synaptic functions due to its gradual conductance switching behavior and high switching ratios, which can reach up to 10^5^.^[^ ^45^ ^]^ To date, a variety of oxide and sulfide‐based semiconductor devices named analog memristors, such as WO_3_, MoS_2_, CeO_2_, etc, have been constructed. Although current scholars have already been able to implement memory and computational functions with memristors, most devices are not detached from the probe table, and the mentioned functions cannot be realized in situ miniaturized, which greatly limits their potential applications. Obviously, an urgent need exists to develop an interactive hybrid system that integrates functionality and balanced performance through miniaturized design and modification that effectively combining sensing and memory.

Here, a Field Programmable Gate Array (FPGA) platform boosted integrated “perception‐memory” system is proposed, which mainly consists of electronic tattoos and digital memristors to realize the signals in situ acquisition and processing, as well as the technological breakthroughs and development prospects are explored. The intentionally designed serpentine electrode structure provides superior stability and resistance to stretching for electronic tattoos. Benefiting from this, the electronic tattoo enables prolonged monitoring of surface electromyography (sEMG), electrocardiography (ECG), electrooculography (EOG), and electroencephalography (EEG) signals. The developed memristor exhibits an exceptionally high switching ratio, reaching 10^4^ at a limiting current (I_CC_) of 100 µA. The multilevel resistive switching behaviors of the RRAM device are investigated by tuning the compliance currents. The integrated system not only includes physiological signal sensing and in situ memory but also enables real‐time interaction with the master to achieve physiological signal recognition. To demonstrate the capabilities of the proposed integrated system in transitioning from electrophysiological signal sensing to intelligent recognition and information memory, two specific applications are explored, specifically including: i) by the acquisition of sEMG signals generated from gestures, assisted by a one‐dimensional (1D) convolutional neural network, a manipulator follows the human hand system in real‐time is realized to facilitate intelligent human‐computer interaction, and the returned results are deposited into the memristor via FPGA, involving the display of the encoded digits memorised. ii) the perception of sEMG signals from the person's face, whose recognized results are utilized to control a smart home system, including emergency call, fan control, and light control. What's more, based on the logical entailment function of the memristor, the on/off control of the music interface is achieved by combining the acquired sEMG signals from the face and gestures. In summary, the integrated “perception‐memory” system proposed in this work provides an effective approach to low‐power intelligent information processing.

## 2 Results and Discussion

### 2.1 Design of the Integrated “Perception‐Memory” System

Inspired by the biological system with physiological signals generation and memory, the integrated “perception‐memory” system consists of an electronic tattoo and digital memristor, as depicted in Figure 1a (right). The proposed memristor, based on the Ag/Tantalum Oxide/Indium Gallium Zinc Oxide/Indium Tin Oxides (Ag/Ta_2_O_5_/IGZO/ITO) structure, is driven by the Digital‐to‐Analog Converter (DAC) for memory and refreshment. The Ta_2_O_5_/IGZO‐based memristors have been selected as memory devices for hybrid systems due to the non‐volatile nature of their resistance and high switching ratios. In the application scenario of manipulator control, the memristor can store the recognized human hand movements. In addition, a hardware‐level logical entailment is constructed based on its memristive property, enabling the on/off control of the music interface by combining it with myoelectric signals. Figure 1b shows the Scanning Electron Microscopy (SEM) image of the functional layer of the designed memristor, which mainly consists of about a 55 nm thick layer of IGZO and a 95 nm thick layer of Ta_2_O_5_. The electronic tattoo is a component that has an Al electrode layer and an Ag electrode layer stacked in succession on the paster, resulting in an Ag/Al/Paster‐based electronic tattoo (AAP). The specially designed serpentine structure of the electronic tattoo provides excellent resistance to stretching, as shown in Figure 1c, which remains stably attached to the skin even when stretched, twisted, or squeezed. Combining the superior sensing performance of the electronic tattoo and the outstanding memory capability of the memristor, the integrated “perception‐memory” system is designed with the framework shown in Figure 1d–(I), which comprises three components: top interaction recognition, control bus, and block driver. By utilizing the AAP's superior perception of sEMG signals, the Analog‐to‐Digital Converter (ADC) is driven to realize the acquisition of bioelectricity (Figure 1d–(II)), and the real‐time interaction with the host via Bluetooth achieves the action recognition. Finally, driven by the voltage management block (Figure 1d–(III)), the memory and switching capabilities of the memristor are achieved, and the control of robot devices and home appliances is realized by FPGA. Figure 1e shows a photograph of the hardware system, with the memristor cross array packaged and mounted on a printed circuit board (PCB). The PCB also contains an ADC, two DACs, multiplexed trans‐impedance amplifiers (TIAs), a memristor array, a Bluetooth module, and an sEMG signal amplification and filtering module, enabling real‐time interaction with the FPGA and personal computer (PC). In addition, the chip enters standby mode when not in use to reduce the overall system power consumption. Among them, the circuit diagram of the memristor driven through the DAC and captured after transimpedance amplification is illustrated in Figure S1 (Supporting Information). Figure S2 (Supporting Information) shows that the sEMG signal acquired is processed through amplification and filtering before being acquired by the ADC and then sent to the PC via the FPGA through the Bluetooth module. Here, the first stage of the amplification and filtering module utilizes an instrumentation amplifier. The amplification is ≈250 times, and the low noise and high input impedance characteristics of the chip make it particularly suitable for amplifying weak signals. Subsequently, after the secondary active high‐pass and low‐pass filtering, the frequency band of the filter is ≈0.7–530 Hz. Finally, after the voltage level is raised to 1.5 V, the FPGA drives the ADC to collect the processed signals and transmits them to the PC via Bluetooth for neural network recognition.

### 2.2 Characterization and Performances of Electronic Tattoos

**Figure 2:**
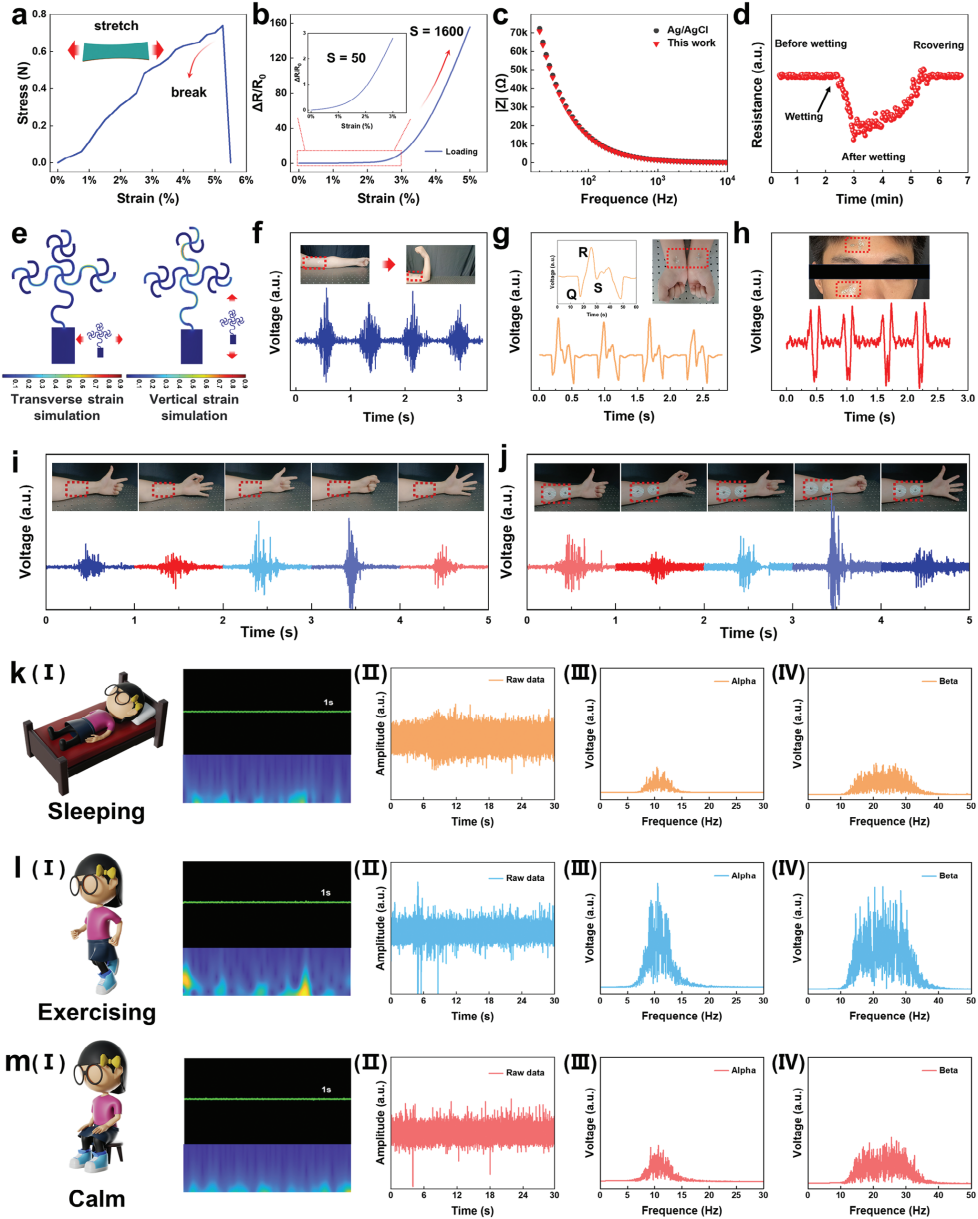
The characterization and performances of AAP and monitoring of physiological signals. a) Strain and stress response of the AAP. b) Stretch response of the AAP. c) The interfacial impedance of the AAP and Ag/AgCl electrodes. d) Restorative monitoring of electrodes. e) Stress simulation in transverse and vertical directions. Electrophysiological signals detection of the AAP, such as f) sEMG signals, g) ECG signals, and h) EOG signals. i) The AAP and j) Ag/AgCl detection of sEMG signals for five different gestures. EEG signals monitoring of k‐(I)) sleep, l‐(I)) exercise, and m‐(I)) calm states. Raw waveforms (k‐(II), l‐(II), m‐(II)) of EEG signals in three states. Extraction of the EEG Alpha waves (k‐(III), l‐(III), m‐(III)). Extraction of the EEG Beta wave (k‐(IV), l‐(IV), m‐(IV)).

Conventional sEMG signal electrodes are uncomfortable to wear due to their non‐deformable properties, which limit the natural deformations of the skin. For this reason, an ultrathin deformable electronic tattoo is designed to overcome the shortcomings present in conventional electrodes. As shown in **Figure** **2**a, the stress versus strain curve of the AAP reveals that the stress increases as the growth of strain. Fracture occurs when the strain reaches 5%, while the stress is capable of reaching 0.7 N, which is sufficient to meet the deformation of the skin due to daily wear. The sensitivity (*S*) of a strain sensor is generally defined as S = (ΔR/R_0_)/ΔL, where *ΔL* represents the change of applied length, *ΔR* denotes the resistance change measured before and after applied stress, and *R*_0_ is the initial resistance. Based on the above equation, Figure 2b depicts the relative change of resistance with strain up to 5% and zooms in on the change at 0–3% strain. It can be seen that when the strain range is from 0 to 3%, the sensitivity change is relatively small, reaching only 50. This benefits from the serpentine structure of the design that protects the device from excessive cracking. With further pressure increases, the sensitivity sharply rises when the strain range exceeds 3%. At deformations reaching close to 5%, the sensitivity reaches 1600, which is attributed to the large stresses that generate excessive cracks, resulting in increased resistance changes. In order to validate that AAP has the ability to capture high quality electrophysiological signals, the AAP and commercially available Ag/AgCl electrodes are attached to the human arm for the analysis of interfacial impedance (Figure 2c). The AAP exhibits an interfacial impedance comparable to that of the commercial electrode Ag/AgCl, and at 50 Hz, the impedance (≈28 kΩ) is lower than the Ag/AgCl (≈30 kΩ). Figure 2d detects the change in AAP resistance when the simulated human body perspires. With the addition of vapor on the surface of the electrode, the resistance value of the AAP initially decreases and then gradually returns to its initial state as the water evaporates, proving its ability to handle multiple occurrences during wear and demonstrating the stability of its performance. Meanwhile, the AAP's perception ability is tested for the same action at different relative humidity and temperature, as shown in Figure S3a,b (Supporting Information). It can be seen that the device demonstrates excellent signal perception in high‐temperature and high‐humidity environments. In addition, the performance of the device is tested under conditions of squeezing, stretching, and twisting, as illustrated in Figure S4 (Supporting Information). Compared to the original state, the noise of the signal is slightly increased due to the change of interface impedance. Ag electrodes are not inherently stretchable, but through structural optimization, the electrodes exhibit some resistance to stretching. The distribution of forces applied to the AAP in the transverse and vertical directions is simulated by COMSOL, as shown in Figure 2e. The AAP generates a 5% stretch in the longitudinal direction. The longitudinal deformation generates a more uniform distribution of forces and provides greater resistance to strain compared to the forces generated by transverse strains. This difference is attributed to the absence of an electrode patch induced in the longitudinal direction. The strain simulation model additionally validates the unique capability of serpentine structures in safeguarding the device against excessive cracking under lower‐stress conditions.

Due to its low interfacial impedance, the AAP is applied to detect human physiological signals. As shown in Figure 2f, the AAP is sequentially applied to one side of the upper arm, and electrophysiological signals resulting from the contraction of muscle tissues are detected when the arm undergoes bending and straightening motions. In four repetitions of the action, the detected waveforms are essentially the same, demonstrating good repeatability. The ECG signal is a synthesis of the electrical activity of the heart on the surface of the body and is an extremely valuable tool in diagnosing heart disease. As shown in Figure 2g, by applying electrodes to the wrists, pulses from both arms can be detected, and the Q, R, and S waves can be used for subsequent analysis of health conditions. Figure 2h demonstrates the ability of the AAP to detect the static EOG signals by applying it to the forehead and lower eyelid. And with distinct peaks and valleys, it can reflect the health information of the eyes. Due to the inhomogeneous and wrinkled nature of human skin, as well as the significant shape changes that occur during physical activity, electrodes with high conductivity and can transmit stable epidermal electromyographic signals are required.

Additionally, Table S1 (Supporting Information) compares the published electronic tattoos and our work. Stable and accurate detection of sEMG signals remains a considerable challenge, both in the laboratory and in the clinic. To evaluate the ability of the AAP as an electrode for sEMG signals, a comparison between a pair of AAP electrodes and a pair of commercial gel electrodes is conducted. A comparison between the AAP and commercial gel electrodes acquiring the same five sets of gesture movements is shown in Figure 2i–j. The AAP exhibits stronger noise immunity because it conforms to the deformation of the skin. On the other hand, the strong driving force between the gel electrode interfaces leads to increased noise interference caused by the delamination of the electrode interfaces. To prove the repeatability of the device, the signal perception of the same action is tested for various samples, as shown in Figure S5a (Supporting Information). It can be seen that AAP exhibits good reproducibility, with signal correlation coefficients of up to 40% between different samples. More importantly, the same device is tested on a long durability line, as shown in Figure S5b (Supporting Information), for the first day, 7th, 14th, and 21st day. It can be observed that the interference increases over time, and by day 21, the signal is completely obscured by noise. This is due to the breakage of the electrode layer on the surface of the ultrathin AAP by repeatedly attaching and removing it on the skin, which results in the blocking of electrode sensing, an increase in interface impedance, and deterioration in signal quality.

EEG signal is a type of signal produced by recording the activity of the brain, which allows it to be used to assist in repairing the body's motor functions and enhancing human‐computer interaction. Nevertheless, EEG signal has been a focus of research in recent years because it is highly sensitive and prone to interference, making it challenging to monitor. The AAP is worn on the forehead of the volunteers to monitor their brain activity during sleep, exercise, and calm states. The EEG signals recorded for 1 s are analyzed by wavelet transform, and the time‐frequency diagrams shown in Figure 2k–(I),l–(I),m–(I) are obtained. From the time‐frequency plots, it can be observed that brain activity is relatively low during sleep and in a state of tranquility. However, after exercise, it can be observed that brain activity becomes more intense. EEG signal has low frequency and can be roughly divided into five categories according to frequency: Delta wave, Theta wave, Alpha wave, Beta wave, and Gamma wave. These waves are found in frequency bands ranging from 0.2 to 3 Hz, 3 to 8 Hz, 8 to 13 Hz, 13 to 32 Hz, and greater than 32 Hz, respectively. Figure 2k–(II), l‐(II), and m‐(II) depict the original waveforms for a duration of 30 s in the three states, and the Alpha waves (Figure 2k–(III),l–(III),m–(III)) and Beta waves (Figure 2k–(IV),l–(IV),m–(IV)) are extracted for each of the three states, respectively. Alpha waves are the optimal brainwave state for learning and thinking and are associated with deep thinking in humans. While a person is deeply contemplating a problem, the body enters a state of deep relaxation. Beta waves are the brainwave state associated with tension, stress, and brain fatigue, and are also linked to a person's attention and anxiety. With excessive intensity in the Beta band, neural activity becomes susceptible to impulsivity, tension, anxiety, and fatigue. Analyzing from the perspective of Alpha waves, the intensity of brain waves is significantly higher in the exercise state compared to the sleep state and the calm state, while the intensity of brain waves in the calm state is slightly higher than that in the sleep state. This indicates that a state of calmness is optimal for thinking, and it is not advisable to transition directly into a learning state immediately after exercise due to heightened brain activity. Furthermore, when analyzing from the perspective of Beta waves, the intensity of brain waves is highest after exercise. This can lead to issues such as fatigue, so it is important to take appropriate rest. The stable recording of EEG signals is due to the intimate contact of the AAP on the skin, regardless of skin stretching/contraction and sweat secretion. The exceptional ability of the AAP to monitor the EEG signal suggests that it is an ideal candidate for future electronic tattoo applications in EEG signal‐based screening for early disease detection and human‐computer interaction.

### 2.3 Characterization and Performances of Memristors

**Figure 3:**
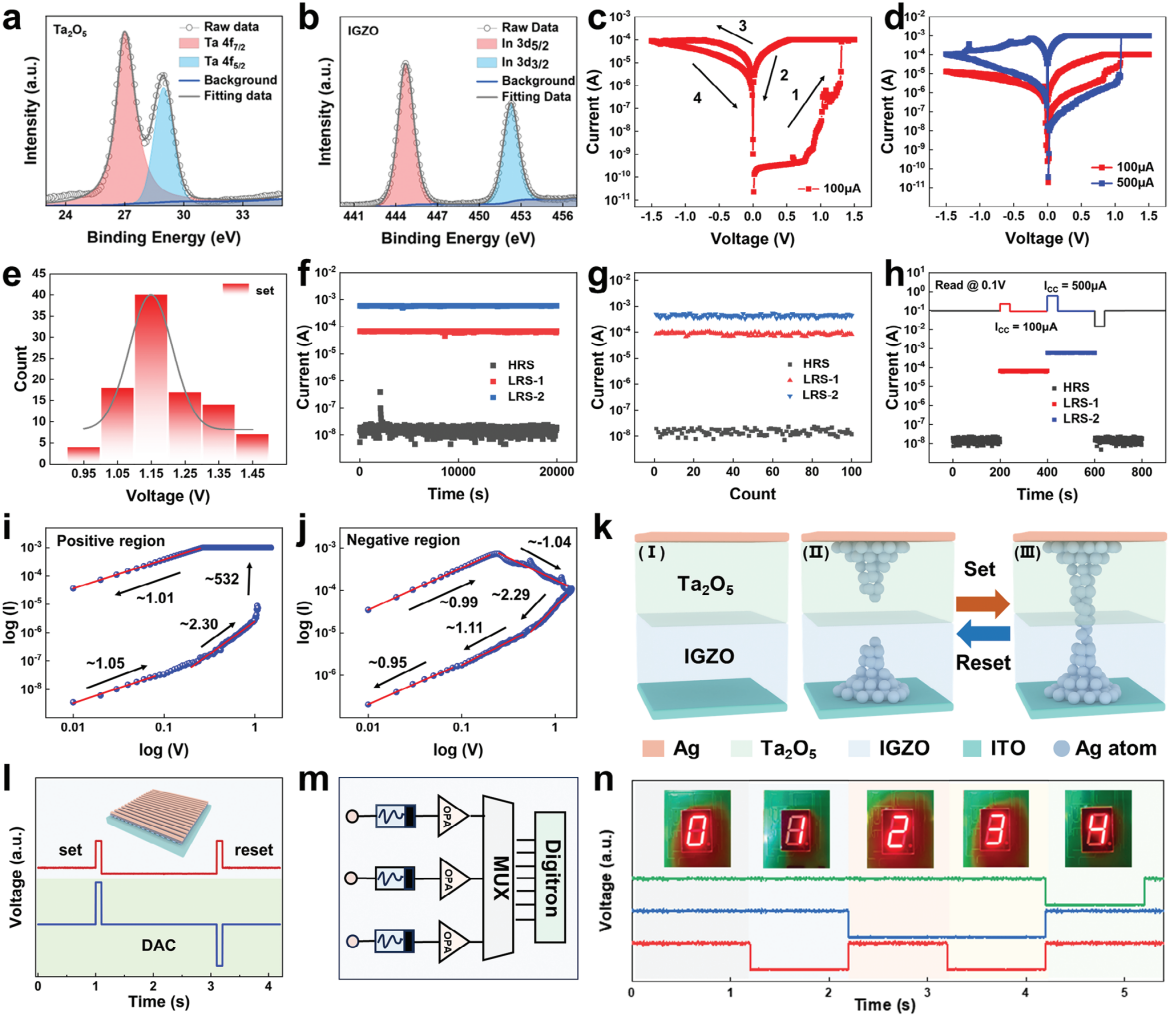
The characterization and performances of the Ta_2_O_5_/IGZO‐based device and the presentation of the display module. The a) Ta 4f and b) In 3d core levels of the Ta_2_O_5_ film and IGZO film. c) Logarithmic *I*–*V* curve of the Ta_2_O_5_/IGZO‐based device. d) Typical *I*–*V* curve of the Ta_2_O_5_/IGZO‐based device under various I_CC_ values (100 and 500 µA). e) Probability distribution of V_SET_, f) time retention test, and g) endurance test under various I_CC_ values. h) State transitions with time under different I_CC_ values. Log (I)‐log (V) plots of the *I*–*V* characteristic curve for the Ta_2_O_5_/IGZO‐based device under i) positive and j) negative sweeping voltages. k) The model of the conduction mechanism of the Ta_2_O_5_/IGZO‐based device. l) DAC signal driving schematic. Insert: Schematic representation of the prepared array. m) The circuit schematic of the display block. n) Signal memory display.

Memory technology is integrated into all aspects of daily life and is an indispensable part of data caching and processing. The resistance value of the digital memristor can remain stable for a long time without external electric field stimulation and has the advantages of low power consumption and non‐volatility over conventional memory devices. In addition, the cross‐array created by the memristor combines storage and logic operations, and this characteristic makes logic operations and data storage be completed in the same device, simplifying system design and enhancing information processing capabilities. Our purpose is to create a memory device utilizing Ta_2_O_5_ and IGZO as active materials that can be stimulated by an electrical signal to achieve a transition of the resistive state, allowing for data storage. Figures S6 and S7 (Supporting Information) show the Atomic Force Microscopy (AFM) images of the prepared Ta_2_O_5_ and IGZO films. The root mean square values of the roughness of the 10 µm × 10 µm Ta_2_O_5_ and IGZO films are computed, resulting in roughness values of 5.575 and 3.902 nm, respectively. In addition, the crystal structures of Ta_2_O_5_ and IGZO films are analyzed by X‐ray Diffraction (XRD), and as seen in Figure S8a,b (Supporting Information), both Ta_2_O_5_ and IGZO films are uncrystallized. Furthermore, the elemental valence states and chemical compositions of Ta_2_O_5_ and IGZO films are characterized by X‐ray Photoelectron Spectroscopy (XPS). **Figure** **3**a shows the XPS spectra of Ta_2_O_5_ films with peaks at 27 and 29 eV that can be attributed to Ta─O bonding. The spin‐orbit splitting of Ta 4f_7/2_ and Ta 4f_5/2_ photoelectrons is found to be ≈2.0 eV. Figure S9a (Supporting Information) shows the XPS spectra of O 1s of Ta_2_O_5_ films, where the spectra can be fitted with two peaks. The main peak, located at 532.5 eV, corresponds to the Ta─O bond, which represents the lattice oxygen, while the smaller peak at 531.2 eV corresponds to the oxygen vacancy (Vo^2+^) in the film. Figure 3b is the XPS spectrum of In 3d of the IGZO film, where the two peaks at 444.7 eV (In 3d_5/2_) and 452.3 eV (In 3d_3/2_) can be attributed to the formation of an In─O chemical bond. In Figure S9b (Supporting Information), the XPS spectra of Ga 3d of the prepared IGZO films show two split peaks at 18 eV (Ga 3d_5/2_) and 19.9 eV (Ga 3d_3/2_), indicating the presence of a stable Ga─O chemical bond. Figure S9c (Supporting Information) shows the XPS spectra of Zn 2p of the IGZO films, and the splitting peaks at 1021.5 eV (Zn 2p_3/2_) and 1044.5 eV (Zn 2p_1/2_) can be attributed to the formation of stable Zn─O bonds. Figure S9d (Supporting Information) shows the O 1s deconvolution peaks of the XPS energy spectra of the IGZO films, indicating that the prepared IGZO films are rich in oxygen vacancies.

The electrical performance of Ta_2_O_5_/IGZO‐based devices is investigated in detail, as shown in Figure 3c, where a series of scanning voltages (0 V → 1.5 V → 0 V → –1.5 V → 0 V) is applied to the silver electrodes, and switching ratios of up to 10^4^ can be achieved when I_CC_ to 100 µA. The initial state of the device is a high‐resistive state (HRS), which gradually transitions to a low‐resistive state (LRS) as the voltage is increased. It transitions to a stable low‐resistive state when it reaches the SET voltage (V_SET_), and the current decreases as the voltage decreases (step 1 → 2). When a negative voltage is applied, it gradually transitions to the HRS with the increase of voltage and eventually returns to its initial state (step 3 → 4). The device is tested for one hundred repetitions at an I_CC_ of 100 µA, as shown in Figure S10 (Supporting Information), from which it can be concluded that the device is capable of consistently switching between high and low grouping states. The electrical properties of the Ta_2_O_5_/IGZO‐based devices are further tested by varying the I_CC_ to 500 µA, as shown in Figure 3d. It is observed from the *I*–*V* curves that the switching ratio increases further up to 10^5^, and the V_SET_ is ≈1.15 V. Subsequently, 100 devices are prepared using the same process, and the probability distribution of the V_SET_ is tested for the same value of I_CC_, which can be approximated and fitted to a Gaussian distribution, as shown in Figure 3e. For an I_CC_ value of 100 µA, the mean value (*m*) of the V_SET_ is ≈1.14 V, and the standard deviation (*σ*) is 0.063. In addition, the coefficient of variation σ/m is further obtained to be ≈5.5%. The smaller value of *σ*/*m* suggests that the V_SET_ distribution is narrower, indicating a more reliable SET process. Figure 3f demonstrates that the device is stable for 2 × 10^4^ s at room temperature environment under different I_CC_ values, indicating its non‐volatility. To assess the reliability of the device, it is subjected to 100 cycles of turning on/off with varying I_CC_. The conductance between the devices exhibits a slight variation, indicating superior reproducibility and electrical characteristics, as depicted in Figure 3g. Furthermore, Table S2 (Supporting Information) compares the published memristors and our work, showing that Ta_2_O_5_/IGZO‐based devices exhibit excellent stability. A series of voltage pulses (1.5 V, 1 ms) is applied to the device to facilitate the continuous writing process. As shown in Figure 3h, the Ta_2_O_5_/IGZO‐based device is HRS in the initial state and transitions to LRS‐1 with the first voltage pulse, with an I_CC_ value of 100 µA. Next, a voltage pulse with an I_CC_ value of 500 µA is applied, causing the Ta_2_O_5_/IGZO‐based device to transition to LRS‐2, and a negative pulse is applied to return the device to HRS.

To investigate the carrier transport mechanism in Ta_2_O_5_/IGZO‐based devices, log‐log fitting is utilized to obtain typical *I*–*V* curves. As shown in Figure 3i, the results from the space‐charge‐limiting current (SCLC) fitting can be evaluated as consistent with ohmic conduction (fitting slope = 1.05).^[^ ^46^ ^]^ As the applied voltage increases, the slope of the fitted plot rises to 2.30, then sharply increases to 532, and finally decreases to 1.01 as the voltage is reduced. Figure 3j illustrates the results of the curve fitting in the negative voltage region, where the slope of the curve is ≈0.99. However, due to the nonlinear change in electron transport and the corresponding change in the energy band structure, it becomes –1.04. As the voltage increases in the negative direction, the slope gradually decreases to 0.95. The conduction mechanism follows the trap‐controlled SCLC model. To further explain the conduction mechanism, the model of the conduction mechanism is depicted in Figure 3k. Figure 3k–(I) shows that the electric field is not applied, and the device is in the HRS. With the application of forward voltage, the ionized silver ions migrate toward the bottom and are reduced to silver atoms (Figure 3k–(II)). When V_SET_ is reached, silver conducting filaments are formed (Figure 3k–(III)), and the Ta_2_O_5_/IGZO‐based device is in the LRS. Subsequently, a reverse voltage is applied, causing the conducting filaments to gradually break and eventually return to the initial state. An array model of the memristor is shown in the insert of Figure 3l, modeled after which a 2 × 6 crossover array is designed and connected to the DAC for driving. A power configuration block is designed inside the FPGA to drive the DAC to perform SET and RESET operations on the Ta_2_O_5_/IGZO‐based device by outputting control signals. As shown in Figure 3l, the bottom half is the DAC output signal and the middle half is the variation of the voltage signal of the memristor actually captured through the ADC, which corresponds to its switching between HRS and LRS. In addition, a memory display block is designed with the circuit structure shown in Figure 3m, where the current passing through the memristor is amplified by an operational amplifier (OPA) and then selected by a multiplexer (MUX) to turn on the digitron. The module connects the three memristors in the prepared array and displays the stored data in real time using encoding to demonstrate that the recognized physiological signals have been successfully saved into the memristors. In the HRS, the current flowing through the memristor is not sufficiently amplified to light the LEDs, and only by switching to the LRS, the current flowing through the memristor is amplified enough to light the LEDs. The HRS is encoded as “0” and LRS is encoded as “1”, which the initial state is HRS, with three bits set to “000” and the module displaying “0”. On this basis, when only the highest bit is set to LRS, it becomes “100”, and the module displays “4”. Figure 3n illustrates the designed display module and device configuration, showcasing the diverse range of applications for Ta_2_O_5_/IGZO‐based memristors in the field of nonvolatile memory.

### 2.4 Applications of the Integrated “Perception‐Memory” System

**Figure 4:**
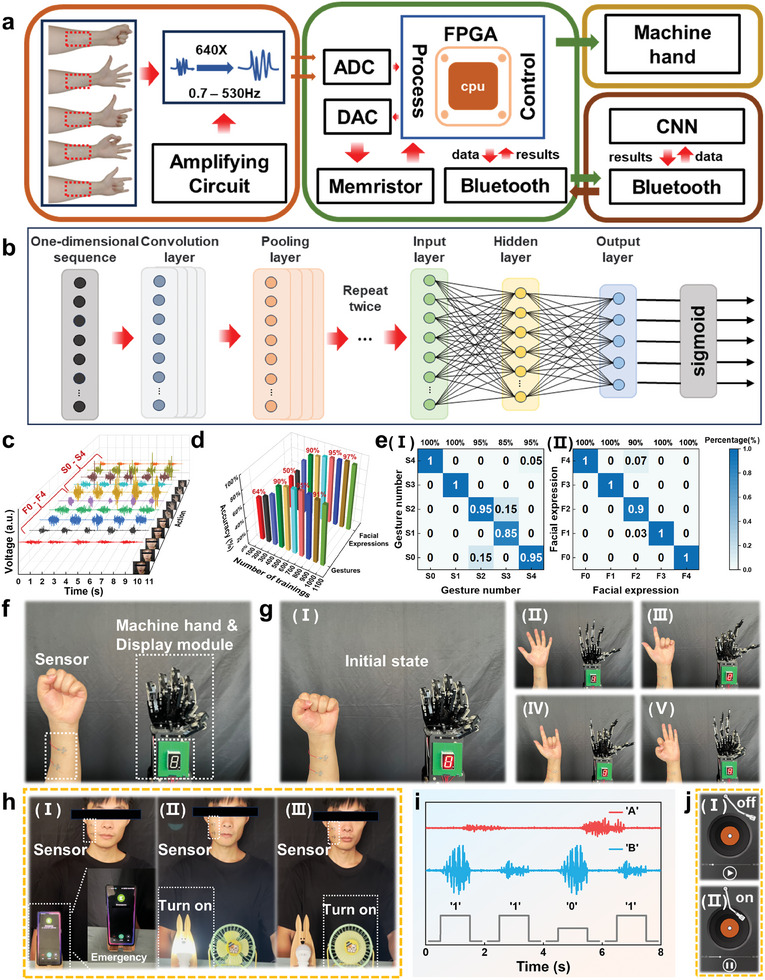
The presentation of the integrated “perception‐memory” hybrid system for manipulator control, emergency calls, and smart homes. a) Workflow of the integrated “perception‐memory” hybrid system based on the AAP and Ta_2_O_5_/IGZO‐based device. b) The framework of neural networks. c) Five facial and gestural 3D signals. d) Neural network training accuracy. e‐(I)) Gestural and (e‐(II)) facial confusion matrixs. f) Overall system presentation. g) The AAP and Ta_2_O_5_/IGZO‐based devices are used for the control of the manipulator and the memory of signals. h) The AAP is used for emergency calls, fan control, and small light control. i) Logic switches composed of combined sEMG and Ta_2_O_5_/IGZO‐based devices. j) On/off control of the music interface.

With the rapid development of the disabled community, ensuring a more convenient and comfortable lifestyle for individuals with disabilities has become an urgent concern. Compared to needle electromyography signals, the detection of sEMG signals has advantages in terms of being painless, non‐invasive, and allowing for long‐term monitoring. Therefore, sEMG signal detection is more practical for the self‐assessment of facial nerve disorders before and after diagnosis in wearable healthcare. The AAP is known for its comfortable fit and excellent ability to detect sEMG signals. Additionally, the prepared memristor offers a high switching ratio and stable electrical characteristics. An integrated “perception‐memory” system has been developed capable of real‐time control of a robotic hand using sEMG signals to simulate the control of a prosthesis by a person with a broken arm and to store the recognized gestures in a prepared Ta_2_O_5_/IGZO‐based memristor. Furthermore, the resistive non‐volatility of the digital memristor is utilized to implement the logical entailment and to control the music interface on and off. The developed “perception‐memory” integrated system is shown in **Figure** **4**a, which includes the AAP, signal processing module, driver and transceiver module, and the terminal device. Figure 4b shows the neural network model designed in the system, and the entire model consists of three convolutional units, one pooling layer, and two fully connected layers. After extracting the 1D sequence composed of sEMG signals using the three convolutional units, the pooling layer spreads the data from all channels into a single dimension with 160 neurons. After two fully connected layers with 20 and 5 neurons, respectively, the final output is passed through an activation function (sigmoid), which determines that the gesture represented by the current data is the one with the highest probability. Five gestures are collected, as shown in Figure S11 (Supporting Information), and labeled as S0–S4. 200 samples of each set of data are shown in Figure 4c (back five waveforms), and the sEMG signals of each hand gesture are more noticeably distinct. Figure 4d (front row) shows sample results of neural network training, with an initial training accuracy of only 60% and a peak accuracy of 95% after 700 training iterations. Figure 4e–(I) shows the confusion matrix depicting the accuracy of the test dataset after training. The accuracy of gesture S0 and gesture S1 is both 100%, while the accuracy of gestures S2, S3, and S4 is 95%, 85%, and 95%, respectively. On one hand, the signal waveform shows a significant similarity between gestures S2 and S3, making it difficult to accurately distinguish between them. On the other hand, the limited number of collected samples can lead to overfitting during training, which decreases the accuracy rate. Figure 4f presents a system demonstration diagram that includes sensors attached to the arm, a robotic hand, and a Ta_2_O_5_/IGZO‐based memristor storage display module. When the gestures of the volunteer change sequentially (Figure 4g–(I) → g‐(II) → g‐(III) → g‐(IV) → g‐(V)), the manipulator gestures with the digits of the display module also change, as shown in Video S1 (Supporting Information). Compared with conventional manipulator control, the proposed hybrid system utilizes the perception and recognition of EMG signals, making it more applicable to a wider population, including individuals with physical disabilities. In addition, overcoming the limitation of a single device being able to sense or recognize only one aspect, the storage of signals enables the analysis and collection of patients’ life data, which helps to provide further personalized medical treatment. More importantly, the memristor maintains its resistive state even when powered down, enabling it to store data for extended periods with low power consumption. The above results successfully demonstrate the effectiveness of the AAP and Ta_2_O_5_/IGZO‐based memristors in myoelectric‐controlled prostheses, providing greater convenience for the disabled.

To further broaden the applications of the AAP and Ta_2_O_5_/IGZO‐based memristor, as well as to assist individuals with disabilities in attaining independence in their daily lives, this work develops a system that utilizes facial sEMG signals to control home appliances and request assistance. In this work, a set of control and call‐for‐help functions for home appliances is proposed through facial sEMG signals. Logical entailment is also introduced by combining sEMG signals and memristor, which ultimately control the activation and deactivation of music. The positions of the zygomatic and musculus risorius muscles on the face are identified and five facial expressions are recorded from the volunteer, whose distribution is illustrated in Figure S12 (Supporting Information). As shown in Figure S13 (Supporting Information), two hundred samples are collected for each facial expression, labeled as F0–F4. Figure 4c (front five waveforms) shows the waveforms of five repeated action acquisitions, which shows that the waveforms of the same facial action are similar, while there are variations between each facial action. After training the neural network, the accuracy rate reaches a maximum of 97% after 900 times, as shown in Figure 4d (back row). From the confusion matrix, it can be seen that the accuracy rate of action F0, action F1, action F3, and action F4 is 100% while the action F2 shows an error between F3 and F0, as shown in Figure 4e–(II). To facilitate daily life and emergency calls, an intelligent control system is designed, including phone dialing, fan control, and light control. In the beginning, the volunteer's face shows no movement, and all devices are turned off. When the volunteer performs action F0, it indicates the need for immediate assistance and will promptly contact the emergency contact, as shown in Figure 4h–(I). When the volunteer performs the actions F1–F4, the small light and fan can be controlled to turn on and off, as shown in Figure 4h–(II),(III) and Video S2 (Supporting Information). To fully utilize the logical entailment function of the Ta_2_O_5_/IGZO‐based memristor, sEMG signals are developed to control the opening and closing of the memory. Figure S14 (Supporting Information) shows the equivalent circuit diagram and truth table for applying V_COND_ (0.75 V) and V_SET_ (1.5 V) to the A and B terminals of the RRAM, respectively, to manipulate the desired output. For logic values, the currents corresponding to HRS and LRS are defined as “0” and “1” respectively. After each voltage operation, the states of the resistors change to A’ and B’, with B’ considered as the output that represents the logic operation. As shown in Figure 4i, the signals for controlling V_COND_ are based on facial expressions F0 and F3, while the control of V_SET_ is based on hand gestures S0,S1. Figure 4j–(I),(II) shows the music interface with embedded logic control when it is logic “1”, the music starts playing and is in a “turn on” state. When the logic is “0”, the music is paused and in the “turn off” state. The integrated “perception‐memory” system successfully proves that the proposed AAP and memristor can assist disabled individuals in leading more convenient and independent lives. It also showcases a wide range of potential applications in the field of myoelectric sensing and memory.

## 3 Conclusion

This work proposes an integrated “perception‐memory” hybrid system with a wearable, comfortable electronic tattoo that closely adheres to the skin and a high switching ratio memristor, incorporated with optimal signal sensing, recognition, and information memory capabilities. To enhance the signal transmission stability of the AAP device and counteract the strain caused by skin deformation, the serpentine sensing layer is skillfully designed, and a structure with multiple electrode layers stacked on top of each other is implemented to improve the tensile strength. It has been proven that the proposed AAP can effectively counteract the stress caused by minor skin deformations and accurately track these deformations to reliably transmit physiological signals. By immobilizing the proposed AAP in various parts of the body, the sEMG, ECG, EOG, and EEG signals were monitored for an extended period. Additionally, the time‐frequency and frequency‐band analyses of the EEG signals were conducted in three different monitored states. What's more, benefiting from the excellent performance of the Ta_2_O_5_/IGZO dielectric layer, the proposed memristor has a high switching ratio and excellent stability. Therefore, a combination of the AAP and Ta_2_O_5_/IGZO‐based memory was utilized to acquire, recognize, store, and demonstrate physiological signals for manipulator control. In addition, facial sEMG signals were captured using the AAP, and exceptional signal‐sensing performance was observed. The facial sEMG signals were used to control the on/off switching of home appliances and the emergency call function, and the logic function was achieved by combining the fabricated memory to control the music interface. The superb functionality of the proposed “perception‐memory” integrated system gives it tremendous potential in the fields of disability, human‐computer interaction, and simulation of the human retina and brain.

## 4 Experimental Section

### Fabrication of the Ag/Al/Paster Film

First, the prepared paster surface was blown with nitrogen gas for backup. Subsequently, a patterned mask plate was applied, and Al and Ag films were sequentially deposited on the pasters using the magnetron sputtering technique. After the chamber pressure was pumped to 5 × 10^−3^ Pa, a 20 sccm flow rate of argon was passed in, and the Al film was prepared by sputtering for 20 min at a working air pressure of 1 Pa with the DC power set to 90 W working on an aluminum target. The Ag film was prepared using the same method and working conditions as described above. The DC power was set to 50 W working on the Ag target, and sputtering was performed for 15 min to prepare Ag films. Finally, the Ag/Al/Paster films were fabricated.

### Fabrication of the Ag/Ta_2_O_5_/IGZO/ITO Film

The customized ITO conductive glass substrates (Yingkou OPV Tech New Energy Co., Ltd) were sequentially cleaned in acetone, alcohol, and deionized water for 10 min and then blown dry by nitrogen gas. Following that, Ta_2_O_5_/IGZO films and crossed Ag electrodes were prepared on ITO conductive glass substrates by magnetron sputtering technique. A resistive layer of Ta_2_O_5_/IGZO was prepared using a square‐hole mask plate measuring 2.5 mm in size. The chamber pressure was pumped to 3 × 10^−3^ Pa, and then argon gas was passed with a flow rate of 30 sccm. The IGZO film was sputtered for 20 min under an operating air pressure of 1 Pa, with the RF power set to 100 W working on the IGZO target. The same compression mold plate and working conditions were used to prepare Ta_2_O_5_ films by energizing a certain ratio of the flow rate of argon and oxygen (30:7 sccm) and heating the substrate to 300 °C. The RF power was set to 100 W working on the Ta_2_O_5_ target and sputtering for 60 min. The Ag top electrode was used to overlay the mask plate onto the bottom electrode ITO, and sputtering was then performed using an argon flow rate of 20 sccm and a DC power of 50 W for 15 min. Finally, Ag/Ta_2_O_5_/IGZO/ITO films were fabricated.

### Characterization and Measurement

The electrical characterization of the devices was measured using a semiconductor parameter analyzer (Keithley 2602B) equipped with a probe measurement system. The cross‐section of the samples was characterized using a Scanning Electron Microscope (SEM Regules800) with an accelerating voltage of 5 kV. The surface morphology, chemical composition, and crystal arrangement of the films were characterized and examined by Atomic Force Microscopy (AFM), X‐ray Photoelectron Spectroscopy (XPS), and X‐ray Diffraction (XRD). The contact resistance of the electronic tattoos was examined using an LCR (E4980AL).

## Conflict of Interest

The authors declare no conflict of interest.

## Author Contributions

Z.Q. and Y.L. conceived this work. Y.L. designed, prepared and tested the devices, developed the integrated “perception‐memory” system and verified the feasibility of this architecture. All authors helped with the experiment design and integrated “perception‐memory” system preparation, and participated in the discussion of experimental results. Y.L. supervised the project. All authors wrote the manuscript, reviewed and commented on the manuscript.

## Supporting information

Supporting Information

Supplemental Video 1

Supplemental Video 2

## Data Availability

The data that support the findings of this study are available from the corresponding author upon reasonable request.
