# Supplementary material for: A Human‐Computer Interaction Strategy for An FPGA Platform Boosted Integrated “Perception‐Memory” System Based on Electronic Tattoos and Memristors
Source: Adv Sci (Weinh). 2024 Jul 24;11(39):2402582. doi: 10.1002/advs.202402582 (PMC11497050; doi:10.1002/advs.202402582)
Supplement: Supplementary file 1 — Supporting Information [file ADVS-11-2402582-s002.pdf]

ADVANCED SCIENCE
Open Access

Supporting Information

for *Adv. Sci.*, DOI 10.1002/advs.202402582

A Human-Computer Interaction Strategy for An FPGA Platform Boosted Integrated "Perception-Memory" System Based on Electronic Tattoos and Memristors

Yang Li, Zhicheng Qiu, Hao Kan*, Yang Yang, Jianwen Liu, Zhaorui Liu, Wenjing Yue, Guiqiang Du, Cong Wang* and Nam-Young Kim**

Supplementary Material

A Human-Computer Interaction Strategy for An FPGA Platform Boosted Integrated "Perception-Memory" System Based on Electronic Tattoos and Memristors

Yang Li, Zhicheng Qiu, Hao Kan*, Yang Yang, Jianwen Liu, Zhaorui Liu,Wenjing Yue, Guiqiang Du, Cong Wang*, Nam-Young Kim**

Prof. Y. Li, Z. Qiu, Prof. H. Kan, Y. Yang, J. Liu, Z. Liu, Prof. W. Yue

Shandong Provincial Key Laboratory of Network Based Intelligent Computing

School of Information Science and Engineering

University of Jinan

Jinan 250022, China

Prof. Y. Li

School of Integrated Circuits

Shandong University

Jinan 250101, China

E-mail: sunny_lee2011@hotmail.com

Prof. N.-Y. Kim

RFIC Centre

Department of Electronics Engineering

NDAC Centre

Kwangwoon University

Seoul 01897, South Korea

Prof. C. Wang

School of Electronics and Information Engineering

Harbin Institute of Technology

Harbin 150001, China

Prof. G. Du

School of Space Science and Physics

Shandong University

Weihai 264209, China**Supplementary Figure**

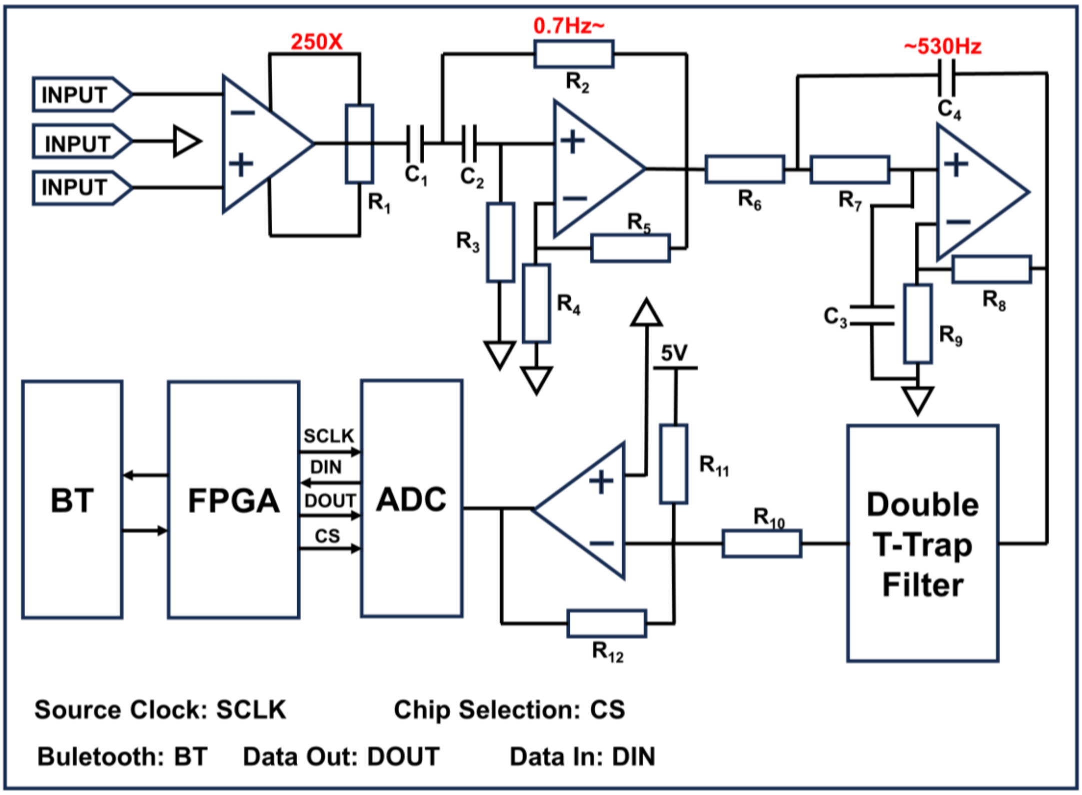

Fig. S1. Circuit diagram for sEMG signal processing.

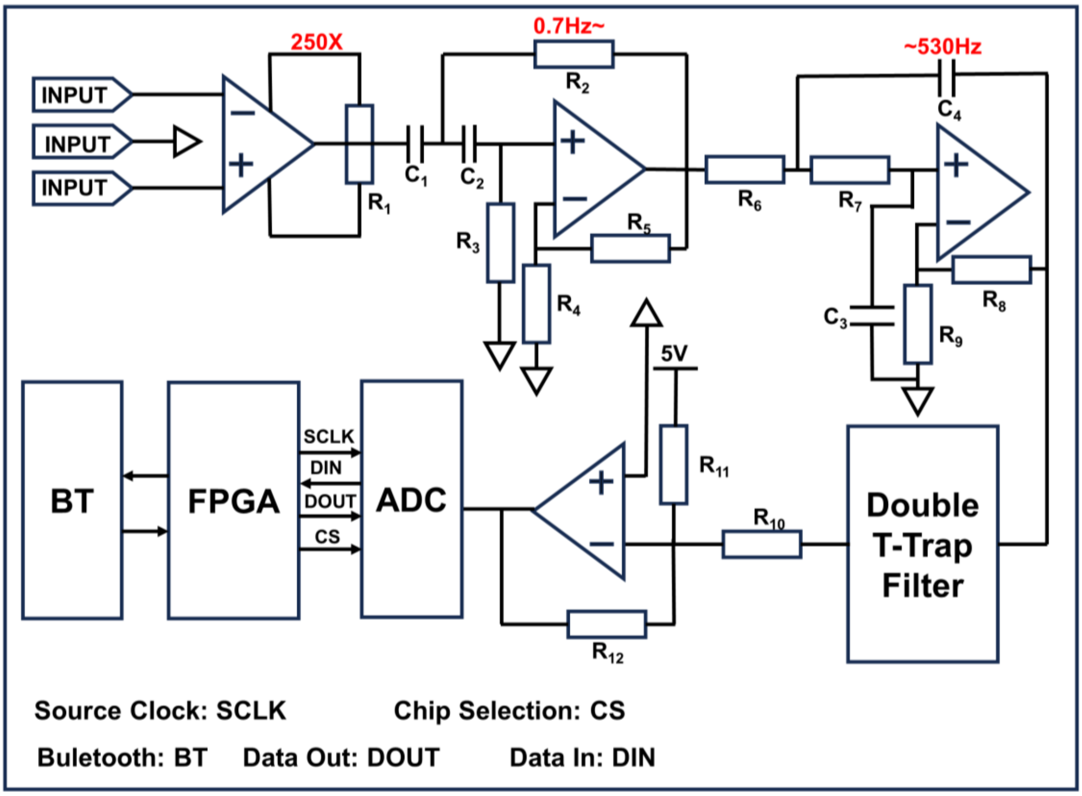

Fig. S2. Circuit diagram for memristor memory and reading.

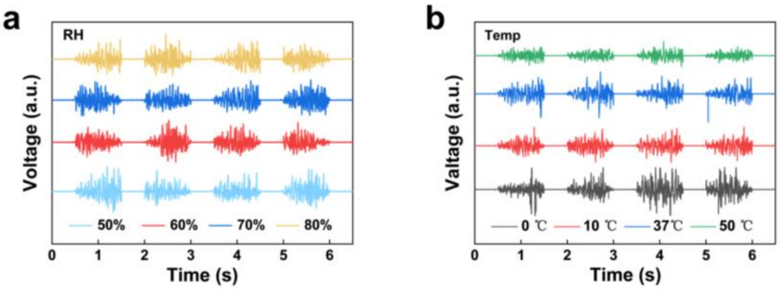

Fig. S3. The influence of humidity (a) and temperature (b) on the perception of AAP signals.

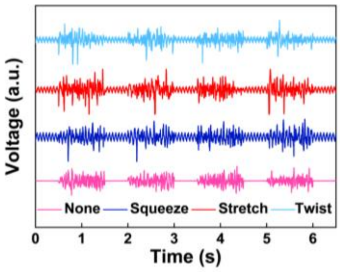

Fig. S4. Testing the AAP's perception of signals under squeezing, stretching, and twisting.

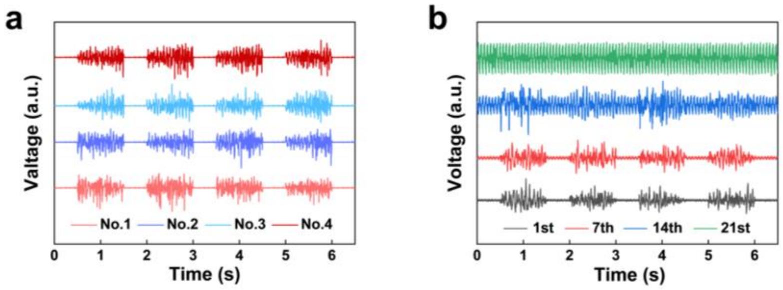

Fig. S5. (a) Tests between different samples. (b) Long-term stability testing of AAP.

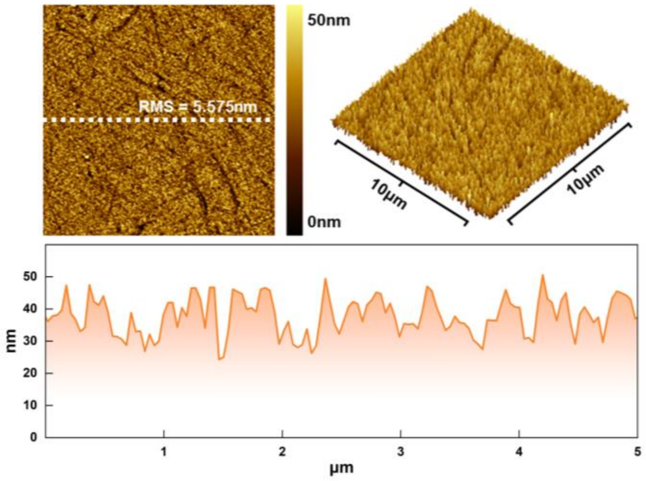

Fig. S6. AFM images (scan size of 10 × 10 μm^2) of the Ta_2O_5 film.

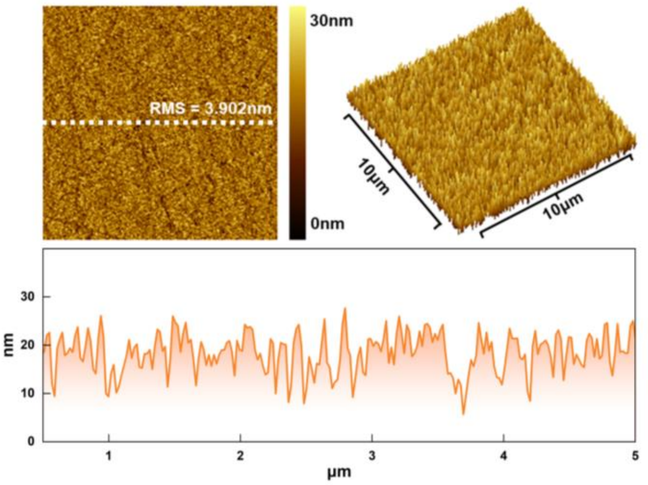

Fig. S7. AFM images (scan size of 10 × 10 μm^2) of the IGZO film.

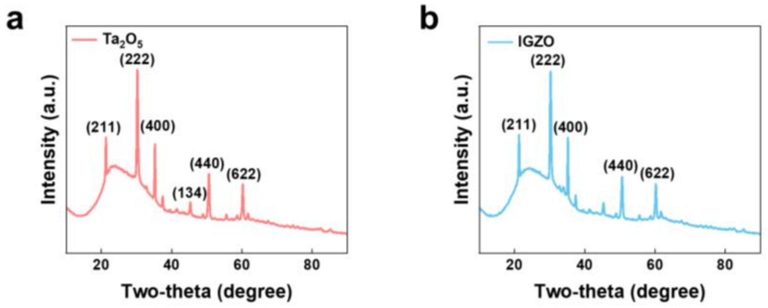

Fig. S8. XRD images of the (a) Ta_2O_5 film and (b) IGZO film.

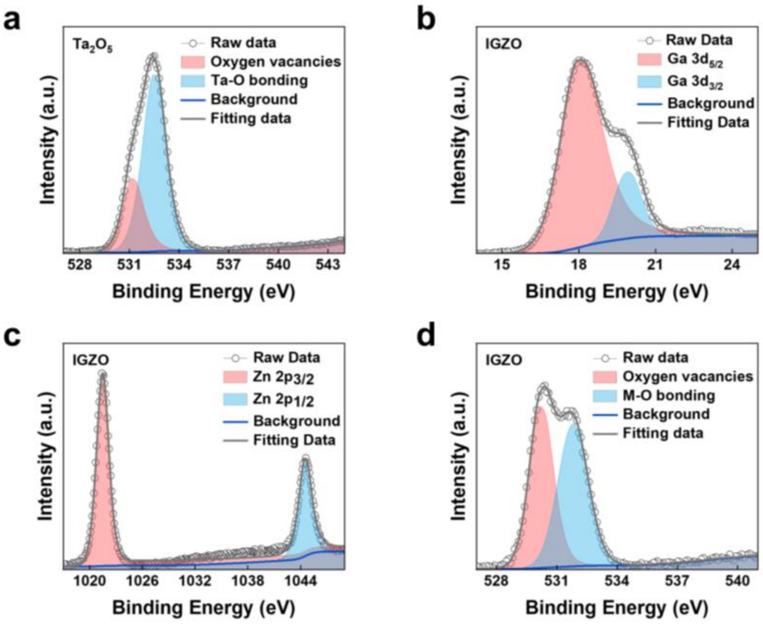

Fig. S9. The (a) O 1s core level of the Ta_2O_5 film. The (b) Ga 3d, (c) Zn 2p, and (d) O 1s core levels of the IGZO film.

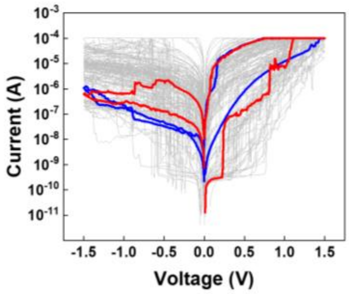

Fig. S10. The run-to-run performance of the Ag/Ta_2O_5/IGZO/ITO device (100 cycles).

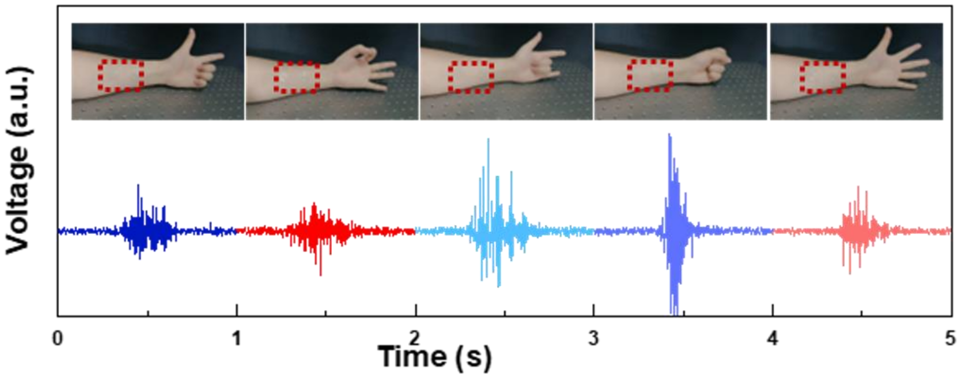

Fig. S11. Presentation of the five gestural sEMG signals.

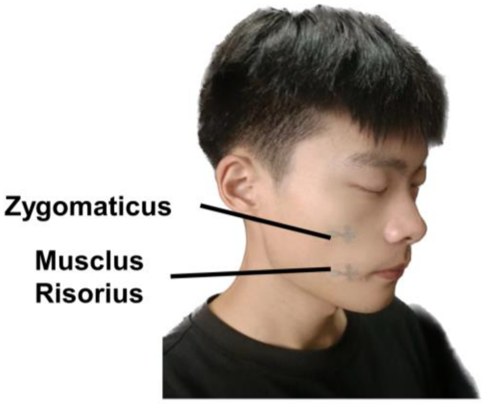

Fig. S12. Schematic representation of facial sEMG signal electrodes.

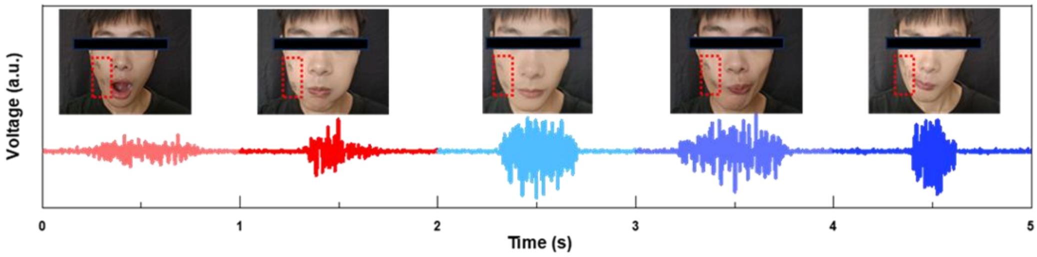

Fig. S13. Presentation of the five facial sEMG signals.

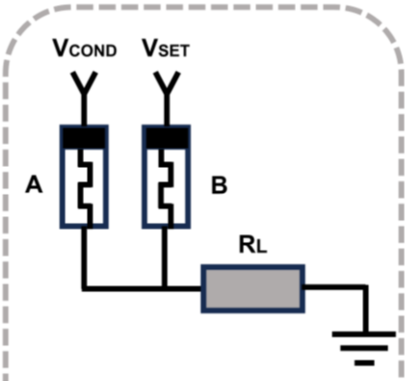

Input		Output
A	B	B’
0	0	1
0	1	1
1	0	0
1	1	1

Fig. S14. IMP logic operation implemented in an RRAM-based circuit and corresponding truth table.

Supplementary Table:

Table S1. Comparison of published electronic tattoos.

Meterials	Stretchability	R/R_0	Thickness	Physiological Signals	Ref.
Ni-EGaIn	-	-	39 μm	√	[1]
Graphite Film	20%	-	200 μm	√	[2]
Au/Cr/PET	-	-	1.5 μm	√	[3]
Au/PET/PVDF/Tegaderm	125%	15	28 μm	√	[4]
PVA/Silk/CNT	-	-	60 μm	√	[5]
MGNF/PI	-	-	3.2 μm	√	[6]
Composite PEDOT: PSS	20%	5	~ 16 μm	√	[7]
Al/P3HT:ICBA/PEDOT: PSS	-	-	1 μm	-	[8]

Au/Ti/PI	-	-	~ 4 μm	√	[9]
Ag/EGaIn	60%	1.5	~ 4 μm	-	[10]
Ag/Al/Paster	5%	160	30 μm	√	This work

Table S2. Comparison of published memristors.

Meterials	Switching Ratio	Time Retention	Multilevel Storage	Cycle Number	Ref.
**2D Tellurium**	> 10	> 10^4 s	-	100	[11]
**Gr/PVA**	~ 500	> 10^4 s	-	150	[12]
**2D h-BN**	> 10	> 10^4 s	-	30	[13]
**Boron Nitride**	~ 10^6	> 3.6 × 10^3 s	-	100	[14]
**PMMA&Mn:$CsPbCl_3$**	~ 10^4	> 10^4 s	√	1000	[15]
**Halide Perovskite**	~ 10^4	-	-	100	[16]
**AlScN/n-GaN**	> 10^5	-	-	-	[17]
**$La_{0.1}Bi_{0.9}FeO_3$/Nb:$SrTiO_3$**	~ 4 × 10^5	> 3.5 × 10^3 s	√	-	[18]
**Silk Fibroin/Graphene Oxide**	~ 10^4	> 10^4 s	√	100	[19]
**IGZO**	~ 10^4	-	-	50	[20]
**Pd/Si:HfO_2/$La_{0.67}Sr_{0.33}MnO_3$/$SrTiO_3$/Si**	~ 176	> 1.3 × 10^4 s	√	100	[21]
**TiN/ZHO/IGZO**	> 10^4	-	-	100	[22]
**Ta_2O_5/IGZO**	~ 10^5	> 2 × 10^4 s	√	100	This work

References

[1] R. Guo, X. Sun, S. Yao, M. Duan, H. Wang, J. Liu, Z. Deng, Adv Materials Technologies 2019, 4, 1900183.

[2] S. Bhattacharya, M. Nikbakht, A. Alden, P. Tan, J. Wang, T. A. Alhalimi, S. Kim, P. Wang, H. Tanaka, A. Tandon, E. F. Coyle, O. T. Inan, N. Lu, Adv Elect Materials 2023, 9, 2201284.

[3] Y. Wang, Y. Qiu, S. K. Ameri, H. Jang, Z. Dai, Y. Huang, N. Lu, npj Flex Electron 2018, 2, 6.

[4] T. Ha, J. Tran, S. Liu, H. Jang, H. Jeong, R. Mitbander, H. Huh, Y. Qiu, J. Duong, R. L. Wang, P. Wang, A. Tandon, J. Sirohi, N. Lu, Advanced Science 2019, 6, 1900290.

[5] S. R. Joshi, A. Pratap, N. Gogurla, S. Kim, Adv Elect Materials 2023, 9, 2201095.

[6] Q.-L. Zhao, Z.-M. Wang, J.-H. Chen, S.-Q. Liu, Y.-K. Wang, M.-Y. Zhang, J.-J. Di, G.-P. He, L. Zhao, T.-T. Su, J. Zhang, X. Liang, W.-L. Song, Z.-L. Hou, Nanoscale 2021, 13, 10798.

[7] Y. Chen, X. Yuan, C. Li, R. Ruan, H. You, Materials 2023, 16, 3499.

[8] B. Burtscher, G. Leising, F. Greco, ACS Appl. Electron. Mater. 2021, 3, 2652.

[9] H. Wang, J. Wang, D. Chen, S. Ge, Y. Liu, Z. Wang, X. Zhang, Q. Guo, J. Yang, IEEE Sensors J. 2022, 22, 3817.

[10] P. A. Lopes, H. Paisana, A. T. De Almeida, C. Majidi, M. Tavakoli, ACS Appl. Mater. Interfaces 2018, 10, 38760.

[11] L. Li, G. Zhang, M. Younis, T. Luo, L. Yang, W. Jin, H. Wu, B. Xiao, W. Zhang, H. Chang, ACS Appl. Electron. Mater. 2024, 6, 2161.

[12] Y. Diao, F. Yang, Y. Jia, M. Su, J. Hu, J. Sun, D. Jiang, D. Wang, Y. Pu, Y. Zhao, B. Sun, ACS Appl. Mater. Interfaces 2024, 16, 2477.

[13] S. Afshari, S. Radhakrishnan, J. Xie, M. Musisi-Nkambwe, J. Meng, W. He, J. Seo, I. Sanchez Esqueda, 2D Mater. 2023, 10, 035031.

[14] S. Wang, X. Liu, H. Yu, X. Liu, J. Zhao, L. Hou, Y. Gao, Z. Chen, Nanomaterials 2024, 14, 327.

[15] Q. Ran, Y. Wang, W. Zhang, N. Xu, W. Chen, X. Tang, J. Phys. Chem. Lett. 2024, 15, 1572.

[16] H. Patil, H. Kim, K. D. Kadam, S. Rehman, S. A. Patil, J. Aziz, T. D. Dongale, Z. Ali Sheikh, M. Khalid Rahmani, M. F. Khan, D. Kim, ACS Appl. Mater. Interfaces 2023, 15, 13238.

[17] M. Liu, S. Lu, Y. Jia, H. Zang, K. Jiang, X. Sun, D. Li, IEEE Electron Device Lett. 2024, 45, 356.

[18] D. Li, X. Zhu, Y. Wu, J. Zhao, K. Zhang, R. Li, D. Hao, Y. Ma, R. Moro, L. Ma, Microelectronic Engineering 2023, 267–268, 111908.

[19] S. Liu, Y. Cheng, F. Han, S. Fan, Y. Zhang, Chemical Engineering Journal 2023, 471, 144678.

[20] F. Qin, Y. Zhang, H. Park, C. S. Kim, D. H. Lee, Z.-T. Jiang, J. Park, K. No, H. Park, H. W. Song, S. Lee, Physica Rapid Research Ltrs 2022, 16, 2200075.

[21] X. Yan, X. Jia, Y. Zhang, S. Shi, L. Wang, Y. Shao, Y. Sun, S. Sun, Z. Zhao, J. Zhao, J. Sun, Z. Guo, Z. Guan, Z. Zhang, X. Han, J. Chen, Nano Energy 2023, 107, 108091.

[22] X. Yan, Z. Zhou, J. Zhao, Q. Liu, H. Wang, G. Yuan, J. Chen, Nano Res. 2018, 11, 1183.

Supplementary Movie

Movie S1. Demonstration of machine hand following control and memory display.

Movie S2. Demonstration of emergency call and smart home control utilizing facial sEMG signals.
